# Collective action and the collaborative brain

**DOI:** 10.1098/rsif.2014.1067

**Published:** 2015-01-06

**Authors:** Sergey Gavrilets

**Affiliations:** 1 Department of Ecology and Evolutionary Biology, University of Tennessee, Knoxville, TN 37996, USA; 2 Department of Mathematics, University of Tennessee, Knoxville, TN 37996, USA; 3 National Institute for Mathematical and Biological Synthesis, University of Tennessee, Knoxville, TN 37996, USA

**Keywords:** social, evolution, mathematical, shared intentionality

## Abstract

Humans are unique both in their cognitive abilities and in the extent of cooperation in large groups of unrelated individuals. How our species evolved high intelligence in spite of various costs of having a large brain is perplexing. Equally puzzling is how our ancestors managed to overcome the collective action problem and evolve strong innate preferences for cooperative behaviour. Here, I theoretically study the evolution of social-cognitive competencies as driven by selection emerging from the need to produce public goods in games against nature or in direct competition with other groups. I use collaborative ability in collective actions as a proxy for social-cognitive competencies. My results suggest that collaborative ability is more likely to evolve first by between-group conflicts and then later be utilized and improved in games against nature. If collaborative abilities remain low, the species is predicted to become genetically dimorphic with a small proportion of individuals contributing to public goods and the rest free-riding. Evolution of collaborative ability creates conditions for the subsequent evolution of collaborative communication and cultural learning.

## 1. Introduction

Our species is unique in a great variety of different ways but the most crucial of them are related to the size and complexity of our brain [1–6]. Brain size in the genus *Homo* tripled in the past 2.5 Myr as a result of several punctuated changes supplemented by gradual within-lineage changes in *Homo erectus* and *Homo sapiens* [2,7,8]. In modern humans, the brain is very expensive metabolically: it represents about 2% of the body's weight but utilizes approximately 20% of the energy consumed [8,9]. Other costs include a need for extended parental care owing to a longer growth period, difficulties at giving birth to larger-headed babies and some mental illnesses that come with brain complexity. A burning question is what factors were responsible for the evolution of human brain size and intelligence despite all these costs.

Two sets of explanations have been debated. Ecological explanations include climate variability and harshness, parasites' and predators' pressure, as well as changes in diet, habitat use and food extraction techniques [10,11]. However, the empirical support for the role of ecology in human brain evolution is relatively weak. Neocortex size does not seem to correlate with several indices related to diet and habitat [12]. There is a statistically significant association of cranial capacity with climate variability and harshness, and parasite pressure, but these factors are much less important than the population density [7].

An alternative set of explanations coming under the rubric of the social brain hypothesis focuses on selective forces resulting from interactions with conspecifics [1,13]. Several types of scenarios have been discussed. One is within-group competition which puts a premium on individuals being able to devise and use ‘Machiavellian’ strategies (including deception, manipulation, alliance formation, exploitation of the expertise of others, etc.) increasing social and reproductive success [6,14]. Comparative studies suggest that species in which Machiavellian-like strategies have been documented have larger brain sizes than related species that do not commonly use these strategies [15]. The plausibility of this scenario is also supported by mathematical modelling [16]. Another scenario emphasizes selection for the ability to maintain social cohesion in large groups (which become increasingly unstable owing to increasing within-group conflicts). It is assumed that larger group sizes are more advantageous because of predatory pressure or in between-group competition. Data do show that brain size correlates with both the group size [12,17] and the population density [7]. The third scenario stresses the advantages of social learning over individual learning under conditions of an increasingly fluctuating environment which was characteristic of the Plio-Pleistocene [18]. Copying the innovations of others through social learning can be advantageous in such environments especially if the population size is sufficiently high [19]. As mentioned above, cranial capacity correlates weakly with environmental variation but strongly with population density [7]. Mathematical models do show that the capacity for social learning can increase when an environment changes in spite of its costs [16,20,21].

Humans are also unique in their innate ability and willingness to cooperate at a variety of different scales [22,23]. Cooperation often requires efficient collaboration with group-mates which is likely to be very cognitively demanding, especially in conditions that require the rapid and fluid coordination of the behaviour of many people, as in hunting or between-group conflict, and the planning for such activities. In fact, it has been argued that the evolutionary roots of human cognition are in our capacity to form shared goals, be committed to them, and collaborate in pursuing them and that this capacity evolved within the context of small-group cooperation [23–25] that enhanced competitive ability vis-à-vis that of other groups [3,5,26]. Within this version of the social intelligence hypothesis, selection for increased ability to collaborate with others (which requires shared goals, joint attention, joint intentions, cooperative communications, etc. [22]) drives the evolution of cognitive abilities. Recent theoretical work has shown that the need for cooperation in dyadic interactions can promote increased brain complexity [27], improved memory [28] and the appearance of tactical deception [29]. However, because of economies of scale, cooperation and collaboration between multiple social partners can result in significantly larger rewards than that in dyadic interactions, and thus could potentially be a very strong selective force for increased social-cognitive competencies.

There are two general types of collective actions in which our ancestors were almost certainly engaged. The first includes group activities such as defence from predators, some types of hunting or food collection, use of fire, etc. The success of a particular group in these activities largely does not depend on the actions of neighbouring groups. I will refer to such collective actions as ‘us versus nature’ games. The second, which I will refer to as ‘us versus them’ games, includes conflicts and/or competition with other groups over territory and other resources including mating. The success of a particular group in an ‘us versus them’ game definitely depends on the actions of other neighbouring groups. The outcomes of both types of games strongly affect individual reproduction as well as group survival.

Collective actions often lead to the collective action problem: if individual effort is costly and a group member can benefit from the action of group-mates, then there is an incentive to ‘free-ride’, i.e. reduce one's effort or withdraw it completely [30–32]. But if enough group-mates follow this logic, the public good is not produced and all group members suffer. Overcoming a collective action problem is a major challenge facing many animal and human groups [33–36]. During the evolution of our species, however, this problem has apparently been solved as humans have strong innate preferences for cooperative and collaborative actions as demonstrated in experiments with small children [25]. My goal here is to answer the following questions: Can the need for within-group cooperation and collaboration in collective actions select for increased cognitive abilities overcoming both the collective action problem and various costs of increased intelligence? If yes, which types of collective actions are most conducive in this regard?

A couple of additional clarifications are in order. First, the models to be considered below focus specifically on the evolution of collaborative ability rather than on the evolution of cognitive abilities in general. The former was preceded by a general increase in brain size throughout the Cenozoic in many mammalian lineages [37]. Greater brain size is expected to correlate with better cognitive abilities. Second, high cognition obviously has other benefits besides the ability to collaborate. These, however, are outside of the scope of this paper. Third, my models focus exclusively on social instincts (encoded in genes) and on deeper evolutionary roots of human social behaviour. As such, they intentionally neglect the effects of language, culture and social institutions which are crucial for human ability to cooperate in very large groups.

## 2. Models and results

I consider a population of individuals living in a large number *G* of groups of constant size *n*. The amount of resources obtained by each group depends on the total effort of its members towards group success; simultaneously, individuals pay fitness costs which increase with their efforts. Group members share the reward equally. Generations are discrete and no overlapping. A group's success in solving the collective action problem controls the probability that the group survives to leave offspring to the next generation. The groups that do not survive are replaced by the offspring of surviving groups. Each individual's effort is controlled genetically and is modelled as a continuous variable. I allow for mutation, recombination, migration and genetic relatedness between individuals. Below I describe the models and main results. The latter are based on analytical approximations and numerical simulations (see ‘Methods’ and the electronic supplementary material). My derivations use the assumption that within-population genetic variation is very low (invasion analysis/adaptive dynamics approximation [38,39]). Numerical simulations show that theoretical conclusions remain valid at a qualitative level even in the presence of genetic variation.

Let *x_ij_* be the effort of individual *i* in group *j* towards the group's success. For members of surviving groups, I define individual fertility as2.1
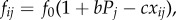
where *b* and *c* are the benefit and cost coefficients, and *f*_0_ is a constant baseline fertility (which can be set to 1 without any loss of generality). As explained below, the probability of the group's success *P_j_* in producing/securing the public good increases with the group efficiency *X_j_*.

A standard practice in evolutionary modelling is to define the group efficiency as an additive function *X_j_* = ∑*_i_x_ij_* of individual efforts. Here I will use a more general and flexible function2.2
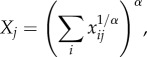
where *α* is a non-negative parameter [40] which I will refer to as collaborative ability.

Collaboration means working with others to achieve shared goals. If individuals are able to collaborate efficiently, a desired outcome can be produced at much smaller individual efforts and/or the same amount of individual effort can result in a much better group outcome than if they acted alone. These intuitions are captured by parameter *α*. In terms of my model, the shared goal is to maximize the group effort *X_j_* which will increase the amount of goods obtained by the group (see below). If collaborative ability *α* is very small 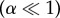, the group is only as efficient as its member with the largest effort (*X_j_* ≈ max*_i_*(*x_ij_*)). Increasing collaborative ability *α* while keeping individual efforts the same increases the group effort *X_j_*. If collaborative ability *α* > 1 (*α* < 1), then the group efficiency *X_j_* is larger (smaller) than the sum of individual efforts. If all group members make an equal effort *x*, then *X_j_* = *n^*α*^x*. The latter function is related to the Lanchester–Osipov model [41,42]. If individuals vary in their efforts but the variation is relatively small, then 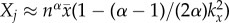, where 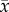 and *k_x_* are the mean and the coefficient of variation of individual efforts *x*. That is, the group efficiency *X_j_* is maximized when the average effort 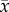 is large but the relative variation in efforts *k_x_* is small, so that there is an increased premium for participation of many individuals.

### 2.1. Us versus nature

I start by treating collaborative ability *α* as a constant, exogenously specified parameter. Consider first the ‘us versus nature’ game. Each group is involved in the production of a public good of value *b* to each member. I define the success probability for group *j* as2.3
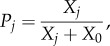
where *X*_0_ is a ‘half-saturation’ parameter (which specifies the group efficiency at which *P_j_* = 50%). The larger *X*_0_, the more group effort *X_j_* is needed to secure the success. I posit that the probability that the group survives to leave the offspring is proportional to its average fertility 
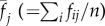
. The groups that do not survive are replaced by the offspring of surviving groups. Specifically, I assume that each group in the current generation descends from a group in the previous generation independently with probabilities proportional to 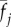. Individuals in each group descend from individuals in their parental group independently with probabilities proportional to *f_ij_*. That is, selection is described by a two-level Fisher–Wright framework common in theoretical studies [43]. Because each group's contribution to the next generation depends on its average fitness, this life cycle corresponds to ‘hard selection’ [44]. This is also a model of multi-level selection [45] where group-level selection favours large efforts *x* (which would increase the probability of group success *P_j_*) while individual-level selection favours low efforts *x_ij_* (which would reduce the individual costs term *cx_ij_*) creating an incentive to free-ride. What makes it beneficial to free-ride is that all group members share equally the benefit of high *P_j_* values independently of their individual contributions to the group's success.

To understand the model's behavior, it is useful to define the parameter *R* = (*b*/*cX*_0_)*n^*α*^*^−1^. Here the first factor is the ratio of the benefit per individual (*b*) and the cost per whole group (*cX*_0_) at a state where the probability of group success *P* = 50%. Under biologically reasonable conditions this ratio will be small. The second factor is an increasing function of *α* which decreases or increases with group size *n* depending on whether *α* < 1 or *α* > 1.

The evolutionary dynamics in this model can be summarized as follows (see electronic supplementary material). If *R* < 1, then at equilibrium individuals make no effort towards the group success (x* = 0). If *R* > 1, then groups make some effort. Specifically, if collaborative ability is low (*α* < *α*_crit_), the population is dimorphic with a great majority of individuals making no efforts and a small proportion (approximately one individual per group) making a substantial collaborative effort. If collaborative ability is high (*α* > *α*_crit_), then all individuals will make positive effort. The critical value 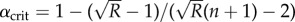 is always smaller than one and approaches one as the group size *n* increases.

The inability of the group to produce the public good when *R* < 1 is a consequence of free-riding exhibited by the group members. In this case, the benefit to cost ratio is too low to secure a positive contribution. If *R* > 1 but collaborative ability *α* is small, the group effort is approximately equal to that of a single individual who is making the largest effort (the ‘strongest link’). Therefore in this case, for the group to be successful it is sufficient to have a single contributing individual per group. In this model, contributors and free-riders differ genetically. (By contrast, in Gavrilets & Fortunato [46], which also predicted groups composed by a mixture of contributors and free-riders, genetic differences were irrelevant but genes were expressed conditionally depending on exogenous factors.) If *R* > 1 and *α* > *α*_crit_, then all individuals start contributing. If group members can effectively collaborate (i.e. *α* > 1), then parameter *R* increases with *n* and the condition *R* > 1 for a positive group effort simplifies. At a state where all group members contribute, the equilibrium individual and group efforts are 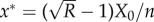 and X* = n^*α*^x*. This shows that higher collaborative ability *α* leads both to a higher individual effort x* (via an increase in *R*) and a disproportionately higher group effort X* (via the synergistic term *n^*α*^*). Effectively with high *α*, the same benefit can be achieved at smaller individual costs, which removes incentives to free-ride.

### 2.2. Us versus them

Next, consider the ‘us versus them’ game. Assume that groups are involved in direct competition for some resources with total value *bG*. Let the share of the resources obtained by group *j* be [47]2.4
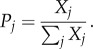
Variable *P_j_* can also be interpreted as a proportion of fights that group *j* won. Losing a conflict can result in group eradication. Assume for simplicity that groups survive to leave offspring to the next generation with probabilities *P_j_*.

The behaviour of this model is strikingly different from that of the first. Now groups always make a positive effort. If collaborative ability is small (i.e. *α* < *α*_crit_ = 1 − 2*n*/((*n*^2^ + 1)(*b* + 1) − 2*nb*)), then the group effort is delivered by a small proportion (roughly 1/*n*) of individuals with the rest making no effort. If collaborative ability is large (i.e. *α* > *α*_crit_), then each individual is making effort x* = (1 + *b*)/*nc*. The collaborative ability parameter *α* does not affect the equilibrium level of effort (although the group efficiency X* will naturally grow with *α*). These differences between the two models are due to the fact that in the ‘us versus nature’ games successful production of a public good requires a sufficiently high absolute group effort, while in the ‘us versus them’ games it is the relative effort that counts, not the absolute. Latter situation creates more favourable conditions for an ‘arms race’ in the individual and group efforts.

### 2.3. Evolving collaborative ability *α*

So far, I have treated collaborative ability *α* as an exogenously specified constant. Increased ability to collaborate results in a more efficient group effort and, thus, one may expect selection for an increased *α*. To investigate this possibility, assume that each individual is characterized by its own, genetically controlled collaborative ability. I will use the average group collaborative ability in computing the group efficiency *X_j_* [48]. To account for individual costs of increased cognitive abilities, I will assume that individual fitness is reduced by a factor 1 − s|*α* − *θ*|, where parameter *s* measures the cost of cognitive abilities and *θ* is a baseline collaborative ability (which can be positive owing to some other benefits of cognitive abilities [3] external to the factors studied here).

To obtain intuition about the strength of forces acting on *α*, assume that the variation in individual efforts *x_i_* is very low. Then in the ‘us versus nature’ games, analyses show (see the electronic supplementary material) that if *x* is very small initially (e.g. maintained by mutation), selection acts against increasing *α*. Selection will act to increase *α* only if individuals make sufficiently large effort in collective action. By contrast, in the ‘us versus them’ games, the level of individual efforts is irrelevant and there will be selection towards a positive value of *α* (unless costs are very high, specifically *s* > ln(*n*)/(*n* − 1). Collaborative ability is predicted to evolve to α* = 1/*s* − (*n* − 1)/ln(*n*). Note that α* does not depend on the costs or benefits of collective action. If the costs are too high, *α* reduces to *θ*. In both types of games, increasing the group size *n* makes the evolution of collaborative ability more difficult.

**Figure 1.**
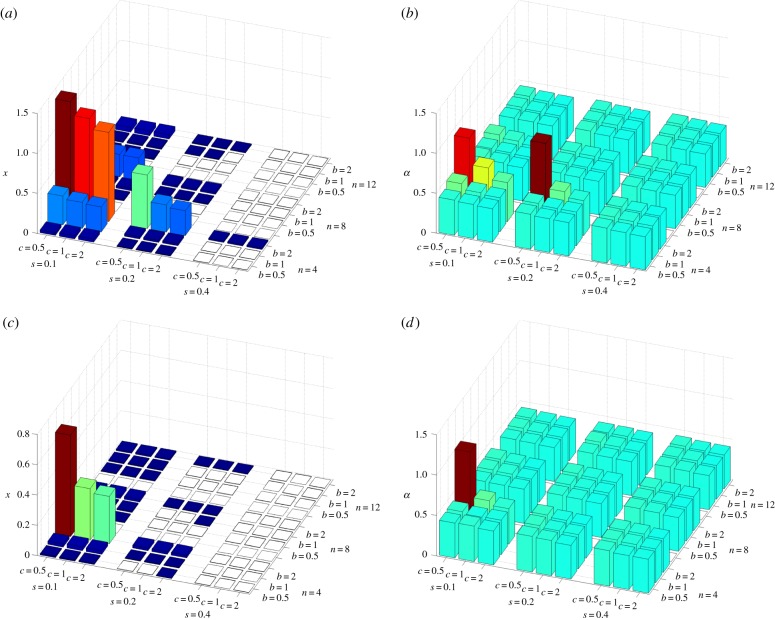
Collective action in ‘us versus nature’ games. Each figure shows the effects of four parameters (benefit of collaboration *b*, cost of individual effort *c*, cost of collaborative ability *s* and the group size *n*) on the average equilibrium values of individual effort *x* (first column) and collaborative ability *α* (second column). (*a*,*b*) Relatively low individual effort is required for the production of public goods (‘half-saturation’ parameter *X*_0_ = 0.25*n*). (*c*,*d*) Relatively high individual effort is required for the production of public goods (‘half-saturation’ parameter *X*_0_ = 0.5*n*). In most cases, individual effort *x* does not evolve and the collaborative ability *α* remains close to the baseline level of *θ* = 0.4. The height of the bars is also reflected in their colour using the jet colormap in Matlab (low values in dark blue and high values in brown).

**Figure 2.**
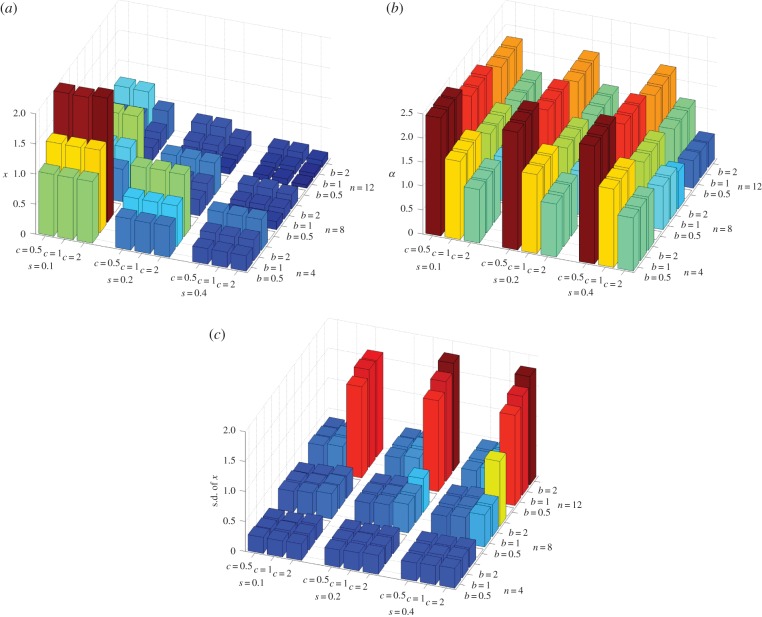
Collective action in ‘us versus nature’ games. Each figure shows the effects of four parameters (benefit of collaboration *b*, cost of individual effort *c*, cost of collaborative ability *s* and the group size *n*) on (*a*) average individual effort at equilibrium *x*, (*b*) collaborative ability *α* and (*c*) within-group standard deviation in individual efforts. The baseline collaborative ability *θ* = 0.4. The height of the bars is also reflected in their colour using the jet colormap in Matlab (low values in dark blue and high values in brown).

**Figure 3.**
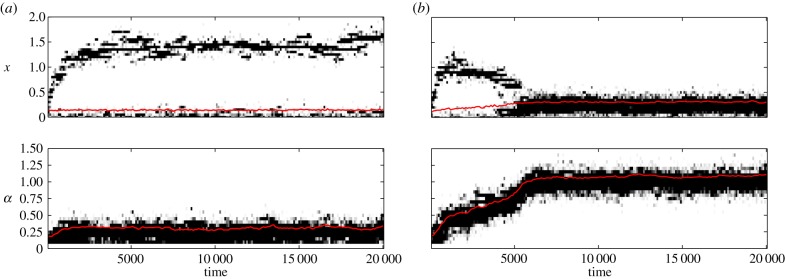
Evolution of individual efforts and collaborative ability in ‘us versus them’ games. (*a*) High cost of collaborative ability *s* (=0.4) results in low collaborative ability *α* and bimodal distribution of individual efforts *x*. (*b*) Intermediate cost of collaborative ability *s* (=0.2) results in the evolution of high collaborative ability *α* and high average efforts *x* . Other parameters: group size *n* = 8, benefit of collaboration *b* = 1, cost of individual effort *c* = 1 and the baseline collaborative ability *θ* = 0.2. The intensity of the black colour is proportional to the number of individuals with the corresponding trait values. Red lines show the mean values.

The conclusions above are based on simple analytical approximations. To check their validity under more general conditions, I have performed individual-based simulations allowing for the joint evolution of individual efforts and collaborative ability (see the electronic supplementary material for details). I assumed that the two traits are controlled by two independent unlinked loci with a continuum of allelic effects. These simulations support my conclusions. In ‘us versus nature’ games, individuals' efforts and collaborative abilities increase only if the costs *c* and *s* and the group size *n* are small, benefit *b* is large, and there is a pre-existing high level of collaboration (i.e. *θ* is high; figure 1; electronic supplementary material). In ‘us versus them’ games, collaborative ability and individual efforts evolve under a much broader range of conditions (figure 2). If collaborative ability does not evolve, then groups become dimorphic as is apparent from increased within-group genetic variation (figure 2*c*). Figure 3 illustrates the difference between these two dynamics.

Overall, my results lead to the following scenario for the evolution of collaborative ability and collective action participation. First, individuals start contributing to collective actions involving direct competition with neighbouring groups of conspecifics (‘us versus them’ games). Subsequently, they evolve improved ability to collaborate in these actions. Once this ability is established at some level, it becomes used in other collective actions. Specifically, individuals start participating in ‘us versus nature’ collective games which then produces additional selection for further increases in collaborative ability and social intelligence. Evolution of collaborative ability creates conditions for the subsequent evolution of collaborative communication and cultural learning [25].

## 3. Discussion

Many social organisms living in stable groups often engage in aggressive group interactions with conspecifics from neighbouring groups over territory and other resources, including mating opportunities [34,35,49–53]. Also, many animals often hunt cooperatively in groups [54,55]. In general, even the most complex types of collaboration in animal groups (e.g. those that include division of labour) do not require advanced cognitive abilities and can emerge from very simple behavioural strategies used by individual group members [56,57]. However, there are limits on the extent and benefits of simple cooperative acts imposed by the collective action problem. As I describe above, evolution of collaborative ability allows groups to mitigate these limits and secure the benefits at much smaller costs.

In the case of cooperative hunting (and other ‘us versus nature’ games), the theory built here predicts a positive group effort only if both the total cost required to secure the benefit and the group size are relatively small. However, unless the collaborative ability is high, this effort will typically be made by a very small proportion of individuals with the rest contributing almost nothing. Collective effort can potentially evolve if group members have cognitive abilities allowing for efficient collaboration, but metabolic and other costs will typically preclude an increase in cognitive and collaborative abilities. In the case of between-group conflict, groups are predicted to always make a positive effort and typically there will be selection for increased collaborative ability. Only if the cost of cognitive abilities is very high, does collaborative ability not evolve and instead the population becomes dimorphic with a small proportion of individuals making a strong effort towards the group's success and remaining group members largely free-riding. If high collaborative ability does evolve through between-group conflict, it can be used in ‘us versus nature’ games which would then further select for increased collaborative ability. This process will lead to a further increase in the effort devoted to ‘us versus nature’ games and the resulting benefits. By contrast, the effort devoted to between-group conflicts does not depend on the collaborative ability and thus will remain stable as it evolves. Realistically low levels of genetic relatedness will not much affect these conclusions (see electronic supplementary material).

Both types of models considered here include individual and group selection as well as public goods production. However in the ‘us versus nature’ games, the success of one group in a public goods production does not affect that of another group. By contrast, in ‘us versus them’ games one group's success means another group's failure. This difference results in stronger selection in the ‘us versus them’ models which in turn produces a larger evolutionary response both in individual contributions and collaborative ability.

The evolution of collaborative ability as studied here requires between-group competition. However, strong competition by itself is not enough. In terms of my models, the crucial factors are (i) the level of baseline collaborative ability, (ii) benefits and costs of collective actions, (iii) costs of collaborative ability, and (iv) presence of relevant genetic variation. In the models, collaborative ability evolves only if these four factors are in an adequate range. One can argue then that somehow in the last couple of million years our species found itself under the right conditions. For example, costs of having a big brain might have been reduced as a result of a shift in diet (‘expensive tissue’ hypothesis, [58]) or there was a shift in the main selective forces from ‘selection by nature’ to ‘selection by conspecifics’ (the ‘ecological dominance’ hypothesis, [1]). My models did not attempt to describe these processes mechanistically but rather captured them in a form of constant parameters.

Starting with Darwin's *The descent of man*, many researchers view between-group conflict and warfare as a potentially important selective factor in shaping many human characteristics ([59–61] but see ref. [62]). In particular, it has been argued that between-group conflict was a driving force in the emergence of many human abilities, biases and preferences (such as cooperation, belligerence, leadership, altruism, parochialism and ethnocentrism) as well as human social norms and institutions [63–66,46]. Alexander [26] argued that the need to succeed in between-group competition would select for increased human cognition and mental abilities; thus allowing for more concerted and effective group actions. Here, I have provided strong theoretical support to these arguments by showing that between-group conflict can select for increased intelligence and cognitive abilities used to coordinate group activities, potentially overcoming both the high costs of large brains and the collective action problem.

It is also generally believed that large-game hunting was very important in human evolution. Success in large-game hunting required the consistent coordinated collaboration of multiple hunters. Alexander [26] argued that collaboration in hunting came first and subsequently created conditions for the evolution of collaboration in between-group conflicts (see also [25]). My results, however, show that the reverse sequence (i.e. collaboration in between-group fighting followed by that in large-game hunting [3]) is more plausible. Collaboration and commitment to a shared goal are also very important in within-group coalitions and alliances which represent an efficient form of within-group competition for reproductive success in a number of mammals including hyenas, wolves, dogs, lions, cheetahs, coatis, meerkats, various primates and dolphins [67–69]. My results suggest that within-group coalitions were preceded and promoted by between-group conflicts. Both these hypotheses still require empirical substantiation. Also it has been argued that human cognitive evolution was driven by selection for cooperative breeding [70]. The latter scenario largely relies on indirect [71] rather than on direct benefits as considered here and therefore is less likely. However, once collaborative ability and shared intentionality are established in the species, the evolution of cooperative breeding is greatly simplified.

The prediction of within-group polymorphism in animals with low collaborative ability and no shared intentionality is supported by an observation that most effort in chimpanzees' group activities is provided by a small number of ‘impact hunters’ and ‘impact patrollers’ [72]. The prediction that humans have evolved a genetic predisposition for collaborative group activities is in line with a consistent observation that human infants are motivated to collaborate in pursuing a common goal [22] and that cooperative acts result in activation of brain regions involved in reward processing, independently of material gains [73]. People cooperate when groups face failure owing to external threats, e.g. harsh environmental conditions or natural disasters [74,75]. However, as predicted by the theory above, cooperation increases dramatically in the presence of direct between-group competition [76–81] to a level that ‘cues of group competition have an automatic or unconscious effect on human behaviour that can induce increased within-group cooperation’ [80]. A variety of other facts and observations about human psychology (e.g. in-group/out-group biases, widespread obsession with team sports and sex differences in the motivation to form and skill at maintaining large competitive groups [3]) strongly support the idea about the importance of between-group conflicts in shaping human social instincts.

The models presented here can be extended and generalized in a number of ways. For example, it is known that the outcome of multi-level selection can depend on the frequency of group reproduction events. In the models presented, group reproduction happens every generation according to the Fisher–Wright scheme. An open question is to what extent the results hold up when group reproduction happens less frequently. Also, one can use alternative functions to represent the strength of the group (equation (2.2)) and the costs of collaborative ability, or introduce variation between groups members in, e.g., their strength or valuation of the reward [46], or assume that individual cognitive abilities affect the costs they pay, etc. Future studies of these and similar modification are needed to increase the generality of this approach and shed additional light on the evolution of human cooperation and cognition.

Decades of intensive work by generations of evolutionary biologists have led to a dramatic increase of our knowledge of how new species arise [82,83]. Time is ripe for a systematic effort to understand the ultimate speciation process—that of our own species [84]. Identifying evolutionary roots for and the dynamics of both human cognitive abilities and cooperative social instincts is a necessary step in getting a better understanding of the origins of our ‘uniquely unique’ species [1].

## 4. Material and methods

In numerical simulations, all individuals were sexual haploids; each deme comprised *n* males and *n* females. Only males contributed to the public good production and paid individual costs. Females carried the genes for the amount of effort and collaborative ability but they were not expressed. Each group in the current generation descended from a group in the previous generation randomly and independently with probability 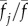 (in the ‘us versus nature’ game) or *P_j_* (in the ‘us versus them’ game). To populate a ‘descending’ group, each female in the corresponding ‘ancestral’ group produced two offspring. The fathers were chosen randomly and independently from the pool of the group's males with probabilities 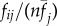. I assumed free recombination between the two genes. The offspring sex was assigned randomly but within each group I enforced an equal sex ratio. Female offspring dispersed randomly between demes while male offspring stayed in the native deme. Simulations ran for 20 000 generations.

In numerical studies of the basic model, I used all possible combinations of the following parameter values: expected benefit per individual *b* = 0.5, 1.0, 2.0; cost-coefficient *c* = 0.5, 1.0, 2.0; group size *n* = 4, 8, 12; baseline collaborative ability *θ* = 0.1, 0.2, 0.4, 0.8, and in ‘us versus nature’ model half-effort parameter *x*_0_ = 0.25, 0.5, 1.0, 2.0. I performed 10 runs for each parameter combination. Some parameters did not change: number of groups *G* = 1000, mutation rate *µ* = 0.001 per gene per generation, standard deviation of the mutational effect *σ*_*μ*_ = 0.1. The initial individual efforts were chosen randomly and independently from a uniform distribution on [0, 0.05]. The initial value of collaborative ability were chosen randomly and independently from a uniform distribution on [*θ*, *θ* + 0.05]. To avoid the appearance of negative fitness values in numerical simulations, I introduced upper boundary on individual efforts *x*_max_ = (1 + *b*)/*c*. I used zero lower boundary on *x*. Electronic supplementary material, figures S1–S5 summarize the results.

## Supplementary Material

Electronic Supplementary Material
