# Supplementary material for: Collective action and the collaborative brain
Source: J R Soc Interface. 2015 Jan 6;12(102):20141067. doi: 10.1098/rsif.2014.1067 (PMC4277098; doi:10.1098/rsif.2014.1067)
Supplement: Electronic Supplementary Material [file rsif20141067supp1.pdf]

Supplementary Information for S. Gavrilets "Collective action and the collaborative brain"

To study my models I used the evolutionary invasion analysis (adaptive dynamics (1, 2)) which focuses on the invasion fitness $w(y|x)$, i.e., the fitness of a rare mutant with trait y in a monomorphic population of individuals with trait x. In the equations describing my main results, I will assume that the number of groups G is large.

Games against nature

Under the assumptions stated in the main text, fitness of individual i from group j is

$$w_{ij} = \frac{\overline{f}_j}{G\overline{f}} \times \frac{f_{ij}}{n\overline{f}_j} \tag{1}$$

where the first and the second ratios are the probability of group survival and the share of the group reproduction going to the individual, respectively, with $\overline{f}$ being the average group fertility in the population (i.e. $\overline{f} = \sum \overline{f}_j / G$).

Additivity. Assume that the group efficiency function is additive, i.e. $X_j = \sum_i x_{ij}$. Then invasion fitness can be written as:

$$w(y|x) = \frac{F}{G\overline{F}} \frac{f}{nF}, \tag{2a}$$

where the mutant's fertility f, the average fertility of the mutant's group F, and the average fertility of groups in the population $\overline{F}$ are

$$f = 1 + bp - cy,\ F = 1 + bp - c\frac{X}{n},\ \overline{F} = 1 + bP - c\frac{\frac{X}{n} + (G-1)x}{G}, \tag{2b}$$

with

$$p = \frac{X}{X + X_0},\ P = \frac{p + (G-1)\frac{nx}{nx+X_0}}{G}, X = y + (n-1)x. \tag{2c}$$

Here, p and P are the probability of success for the mutant's group and the average probability of success in the population, respectively, and X is the efficiency of the mutant's group.

Making a variable change $z = x/x_0$ with $x_0 = X_0/n$, we find that the selection gradient

$$\frac{\partial w(y|x)}{\partial y}|_{y=x} \sim R - (z+1)^2$$

where $R = \frac{b}{cX_0}$. I conclude that if $R > 1$, the individual effort evolves to an equilibrium value

$$x^* = x_0(\sqrt{R} - 1). \tag{3a}$$

This equilibrium is stable. If $R < 1$, then $x^* = 0$.

Synergicity. Assume that the group efficiency function is

$$X_j = \left(\sum_i x_{ij}^{1/\alpha} \right)^{\alpha}. \tag{3b}$$

This assumption results in the equations for P and X becoming

$$P = \frac{p + (G-1)\frac{n^{\alpha}x}{n^{\alpha}x + X_0}}{G}, X = \left(y^{1/\alpha} + (n-1)x^{1/\alpha} \right)^{\alpha}.$$

Using the same approach as above, one finds that the equation for R becomes

$$R = \frac{b}{cX_0}\, n^{\alpha - 1}, \tag{3c}$$

while the nonzero equilibrium value of x is still given by expression (3a). However this equilibrium is stable only if

$$\alpha > \alpha_{\text{crit}} = 1 - \frac{\sqrt{R} - 1}{\sqrt{R}(n+1) - 2}. \tag{4}$$

If the above condition is not satisfied, x^* is a branching point (1, 2) and the population becomes dimorphic with most individuals contributing nothing and on average one individual per group making a large nonzero contribution.

Evolution of α. To study the effects of selective forces acting on collaborative ability α, we assume that individuals are monomorphic with respect to their effort x. I also assume that viability decreases linearly with collaborative ability. Then the fitness of a rare mutant with collaborative

ability β in a resident population with ability α can be written as:

$$w(\beta|\alpha) = \frac{F}{G\overline{F}}\,\frac{f}{nF}, \tag{5a}$$

where the mutant's fertility f, the average fertility of the mutant's group F, and the average fertility of groups in the population $\overline{F}$ are

$$f = (1+bp-cx)(1-s\beta),\; F = (1+bp-cx)(1-s\hat{\alpha}),\; \overline{F} = \frac{F+(G-1)(1+bP-cx)(1-s\alpha)}{G}, \tag{5b}$$

with

$$p = \frac{n^{\hat{\alpha}}x}{n^{\hat{\alpha}}x+X_0},\; P = \frac{n^{\alpha}x}{n^{\alpha}x+X_0}, \hat{\alpha} = \frac{\beta+(n-1)\alpha}{n}. \tag{5c}$$

Here, p and P are the probability of success for the mutant's group and for a group with no mutant, respectively, and $\hat{\alpha}$ is the collaborative ability of the mutant's group. We also assume for simplicity that $\theta = 0$.

Computing the selection gradient $\frac{\partial w(\beta|\alpha)}{\partial \alpha}|_{\beta=\alpha}$ we find that if individual effort $x = 0$, then the selection gradient is $-\frac{s}{1-s\alpha}$ so that α will decrease to zero. This suggests that collaboration effort will experience positive selection only if x is sufficiently large.

Games against other groups

Under the assumptions stated in the main text, fitness of individual i from group j is

$$w_{ij} = P_j \times \frac{f_{ij}}{n\overline{f}_j} \tag{6}$$

Additivity. Assume that the group efficiency function is additive, i.e. $X_j = \sum_i x_{ij}$. Then invasion fitness (i.e. fitness of a rare mutant with trait y in a resident population with trait x) can be written as:

$$w(y|x) = p\,\frac{f}{nF}, \tag{7a}$$

where the mutant's fertility f, the average fertility of the mutant's group F, and the probability of success p for the mutant's group are

$$f = 1 + bGp - cy,\ F = 1 + bGp - c\frac{Y}{n},\ p = \frac{Y}{Y + (G-1)X}, \tag{7b}$$

with

$$X = nx, Y = y + (n-1)x. \tag{7c}$$

Here, X and Y are the group efficiencies for the resident and mutant groups, respectively.

Computing the selection gradient, one finds that x evolves towards a stable equilibrium which, in the limit of large G, can be written as

$$x^* = \frac{1+b}{nc}. \tag{8}$$

Synergicity. Assume that the group efficiency function is given by equation (3b). Then the expressions for X and Y above take form

$$X = n^{\alpha}x,\ Y = \left(y^{1/\alpha} + (n-1)x^{1/\alpha}\right)^{\alpha}$$

Computing the selection gradient I find that there is still an equilibrium at $x^* = (1+b)/(nc)$. However, this equilibrium is stable only for α large than

$$\alpha_{\text{crit}} = 1 - \frac{2n}{(n^2+1)(b+1) - 2nb}. \tag{9}$$

Note that α_{crit} approaches zero from below as n and/or b increase. For $\alpha < \alpha_{\text{crit}}$, x^* is a branching point (1, 2). Numerical simulations show that in these case, the population becomes dimorphic, with most individuals contributing nothing and on average 1 individual per group making a large nonzero contribution.

Evolution of α. Assume first that the population is monomorphic with respect to x. The invasion fitness of a rare mutant with collaborative ability β in a resident population with ability α

can be written as:

$$w(\beta|\alpha) = p\frac{f}{nF}, \tag{10a}$$

where the mutant's fertility f and the average fertility of the mutant's group F are

$$f = (1 + bGp - cx)(1 - s\beta),\; F = (1 + bGp - cx)(1 - s\hat{\alpha}) \tag{10b}$$

with

$$p = \frac{n^{\hat{\alpha}}x}{n^{\hat{\alpha}}x + (G-1)n^{\alpha}x} \tag{10c}$$

being the probability of the mutant's group survival. We assume for simplicity that $\theta = 0$. Computing the selection gradient, one finds that the evolutionary dynamics of the collaborative ability α do not depend on the individual effort x, and that the collaborative ability is predicted to evolve to

$$\alpha^* = \frac{1}{s} - \frac{n-1}{\ln(n)}. \tag{11a}$$

This value is positive if

$$s < \frac{\ln(n)}{n-1}. \tag{11b}$$

Assume next that the population is dimorphic so that on average only m out of n group members make effort x while the remaining group members make no effort. The equations for the invasion fitness stay the same except that probability p of the mutant group success becomes

$$p = \frac{m^{\hat{\alpha}}x}{m^{\hat{\alpha}}x + (G-1)m^{\alpha}x}.$$

I find that the equilibrium value of α becomes

$$\alpha^* = \frac{1}{s} - \frac{n-1}{\ln(m)}. \tag{12}$$

This value is positive if

$$s < \frac{\ln(m)}{n-1}. \tag{13}$$

Thus, decreasing the number m of contributing group members, makes the evolution of collaborative ability more difficult.

Comment. The "us vs. nature" and "us vs. them" games as modeled here differ not only in the production function P_j used (eq. 3 and 4 of the main text) but also in the probability of group survival which was $\frac{\overline{f}_j}{G\overline{f}}$ and P_j, respectively. An interesting question for future research is how the results would be affected if the functions specifying the probability of group survival were the same.

Relatedness

The results above assume that the groups are formed randomly each generation, implying that group members are genetically unrelated. Evolution of cooperation in public goods games studied here is driven by overlapping interests and does not require genetic relatedness (or reciprocity or punishment). Genetic relatedness does however increase cooperation (3, 4). For situations when all individuals contribute in the "us vs. nature" game, the composite parameter R is increased by a factor of $1+r(n-1)$, where r is the average within-group relatedness. This will increase both the range of parameter values resulting in nonzero group efforts and that effort itself. In the "us vs. them" game, each individual effort increases by a factor of $\frac{1+(n-1)r}{1+\frac{n-1}{n}r}$. For example, let female offspring disperse randomly between groups while the male offspring stay in their native group (as in chimpanzees and, likely, our ancestors (5, 6)). Then males within a group will be genetically related with $r=\frac{1}{3(n-1)}$ (7). This will result in about a 30% increase in all individual efforts (assuming that $n \geq 5$).

Details of numerical simulations

All individuals were sexual haploids; each deme comprised n males and n females. Only males contributed to the public good production and paid individual costs. Females carried the genes for the amount of effort and collaborative ability but they were not expressed. Each group in the current generation descended from a group in the previous generation randomly and independently with probability $\overline{f}_j/\overline{f}$ (in the "us vs. nature" game) or P_j (in the 'us vs. them" game). To populate a "descending" group, each female in the corresponding "ancestral" group produced two offspring. The fathers where chosen randomly and independently from the pool of the group's males with

probabilities $f_{ij}/(n\overline{f}_j)$. I assumed free recombination between the two genes. The offspring sex was assigned randomly but within each group I enforced an equal sex ratio. Female offspring dispersed randomly between demes while male offspring stayed in the native deme. Simulations ran for 200,000 generations.

In numerical studies of the basic model I used all possible combinations of the following parameter values: expected benefit per individual $b = 0.5, 1.0, 2.0$; cost coefficient $c = 0.5, 1.0, 2.0$; group size $n = 4, 8, 12$; base-line collaborative ability $\theta = 0.1, 0.2, 0.4, 0.8$, and in "us vs. nature" model half-effort parameter $x_0 = 0.25, 0.5, 1.0, 2.0$. I performed 10 runs for each parameter combination. Some parameters did not change: number of groups $G = 1000$, mutation rate $\mu = 0.001$ per gene per generation, standard deviation of the mutational effect $\sigma_\mu = 0.1$. The initial individual efforts were chosen randomly and independently from a uniform distribution on $[0, 0.05]$. The initial value of collaborative ability were chosen randomly and independently from a uniform distribution on $[\theta, \theta + 0.05]$. To avoid the appearance of negative fitness values in numerical simulations, I introduced upper boundary on individual efforts $x_{\max} = (1+b)/c$. I used zero lower boundary on x. Figures S1-S5 summarize the results.

References

[1] Geritz, S. A. H., Kisdi, E., Meszéna, G. & Metz, J. A. J. Evolutionary singular strategies and the adaptive growth and branching of the evolutionary tree. *Evolutionary Ecology* **12**, 35–57 (1998).

[2] Waxman, D. & Gavrilets, S. Target review: 20 questions on adaptive dynamics. *Journal of Evolutionary Biology* **18**, 1139–1154 (2005).

[3] Nowak, M. *Evolutionary dynamics* (Harvard University Press, Harvard, 2006).

[4] McElreath, R. & Boyd, R. *Mathematical models of social evolution. A guide for the perplexed* (Chicago University Press, Chicago, 2007).

[5] Copeland, S. R. *et al.* Strontium isotope evidence for landscape use by early hominins. *Nature* **474**, 7678 (2011).

[6] Laluenza-Fox, C. *et al.* Genetic evidence for patrilocal behavior among Neanderthal groups. *Proceedings of the National Academy of Sciences USA* **108**, 250–253 (2011).

[7] Gavrilets, S. Human origins and the transition from promiscuity to pair-bonding. *Proceedings of the National Academy of Sciences USA* **109**, 9923–9928 (2012).

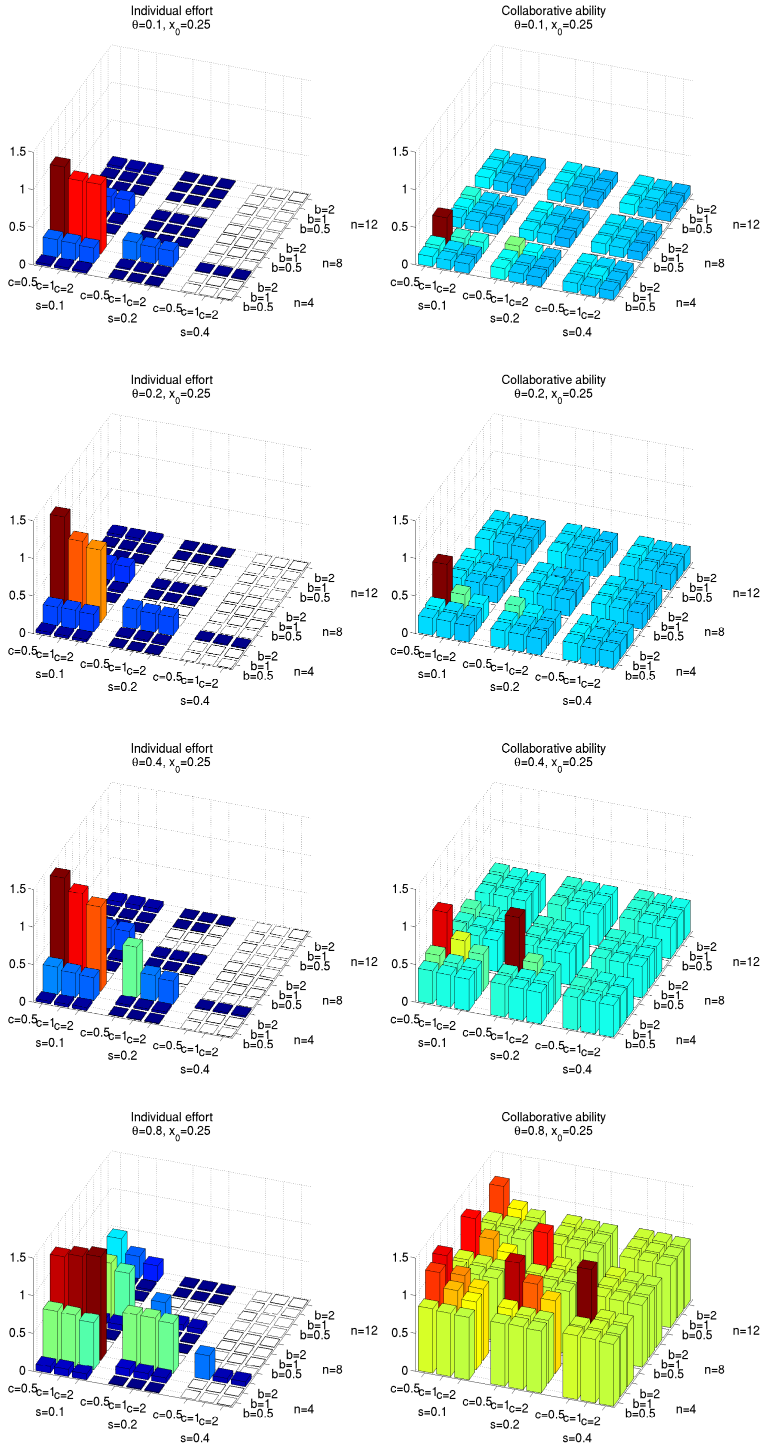

Figure 1: Collective action in "us vs. nature" games with $x_0 = 0.25$.

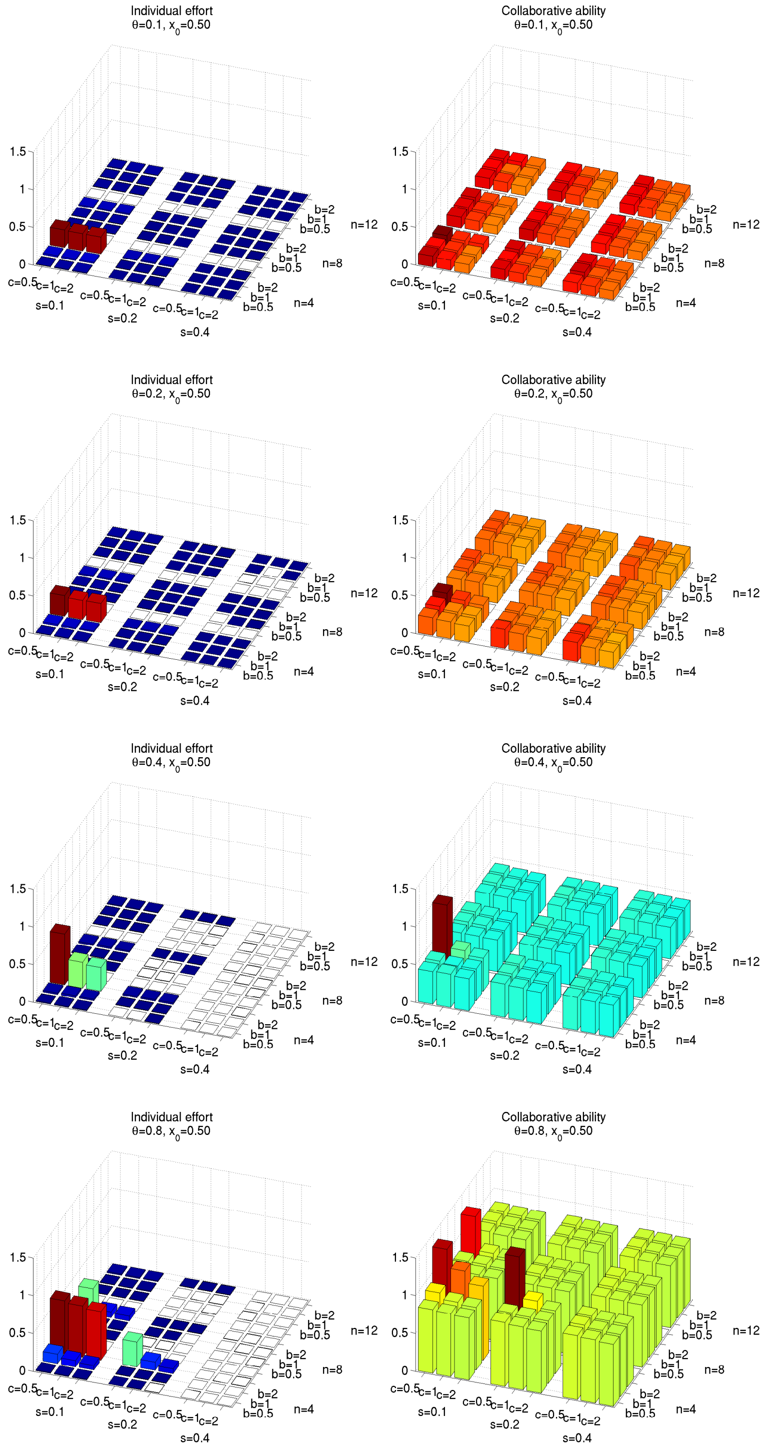

Figure 2: Collective action in “us vs. nature” games with $x_0 = 0.5$.

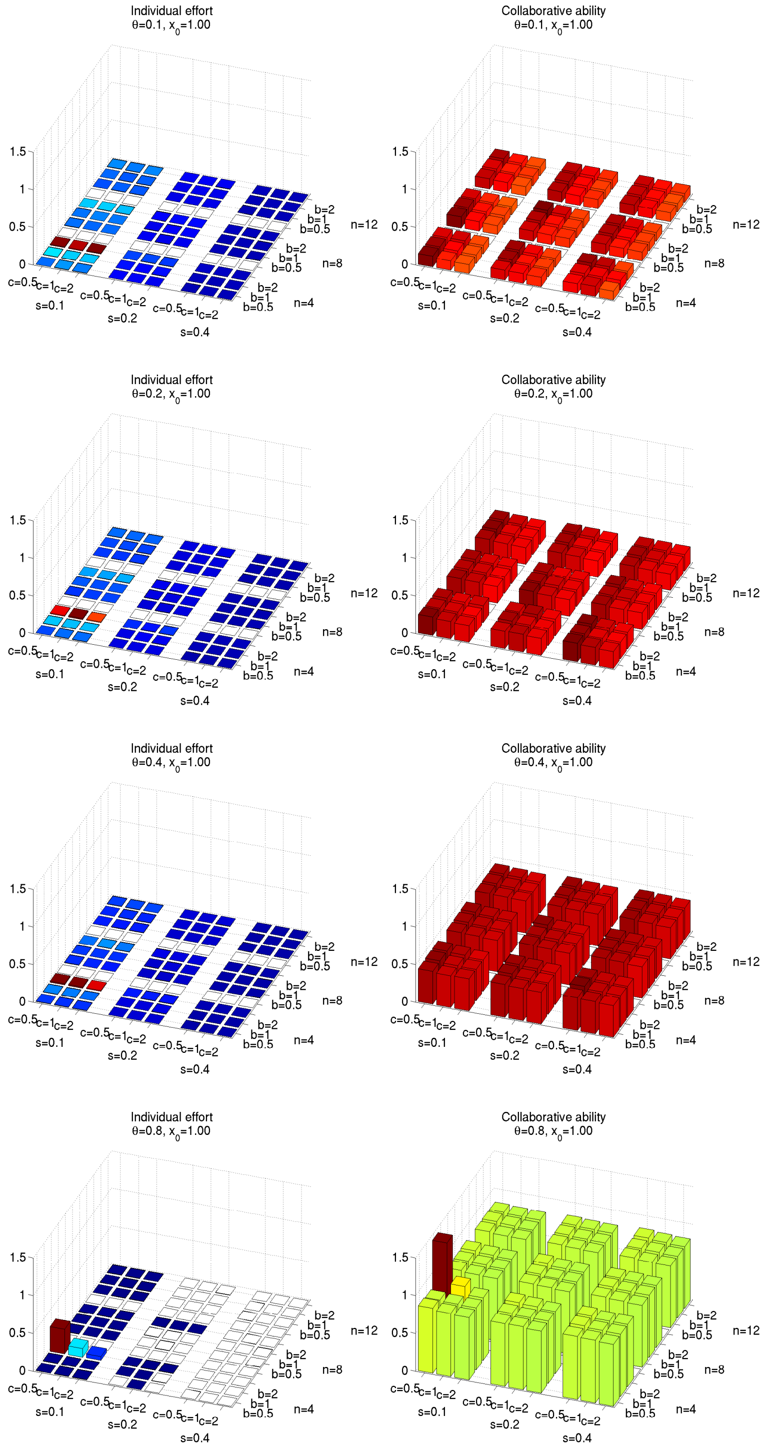

Figure 3: Collective action in “us vs. nature” games with $x_0 = 1$.

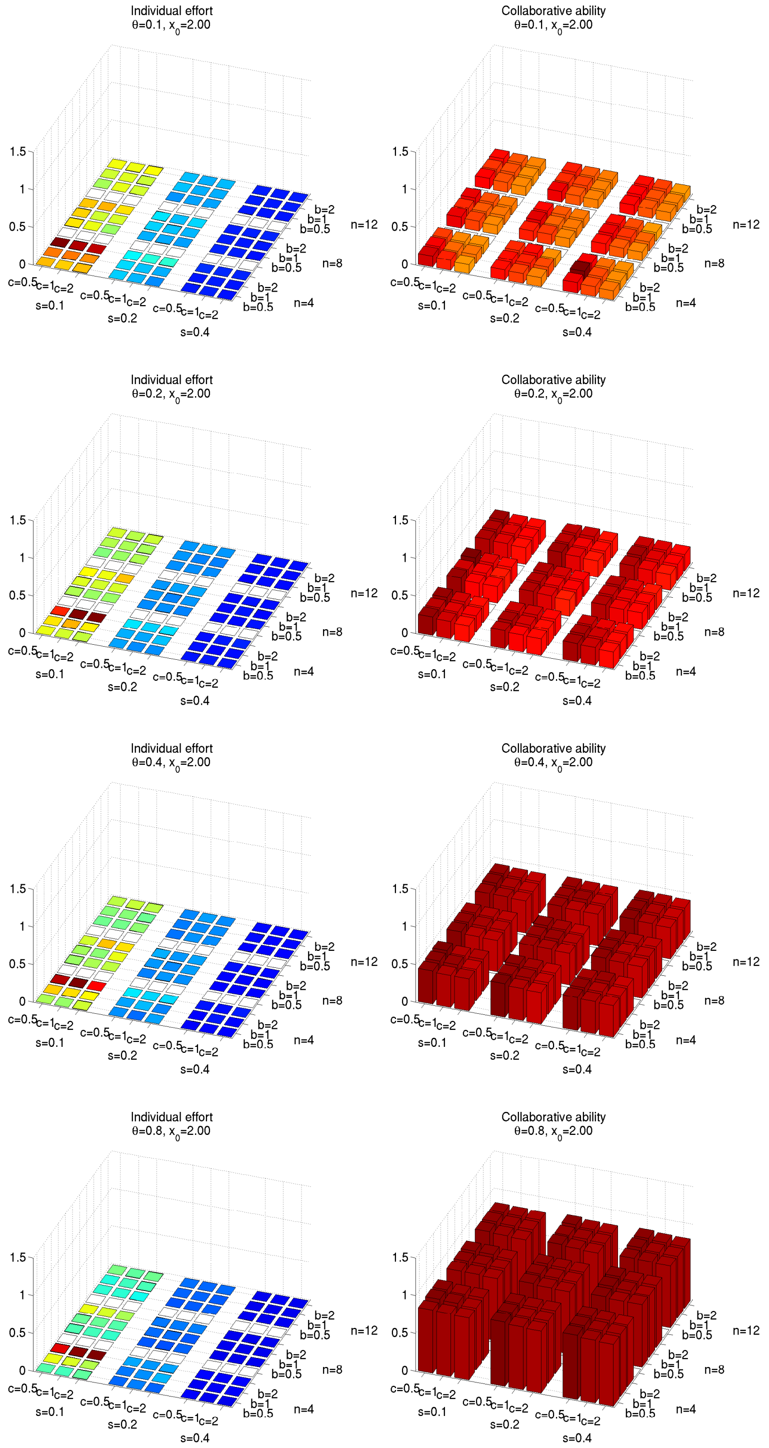

Figure 4: Collective action in "us vs. nature" games with $x_0 = 2$.

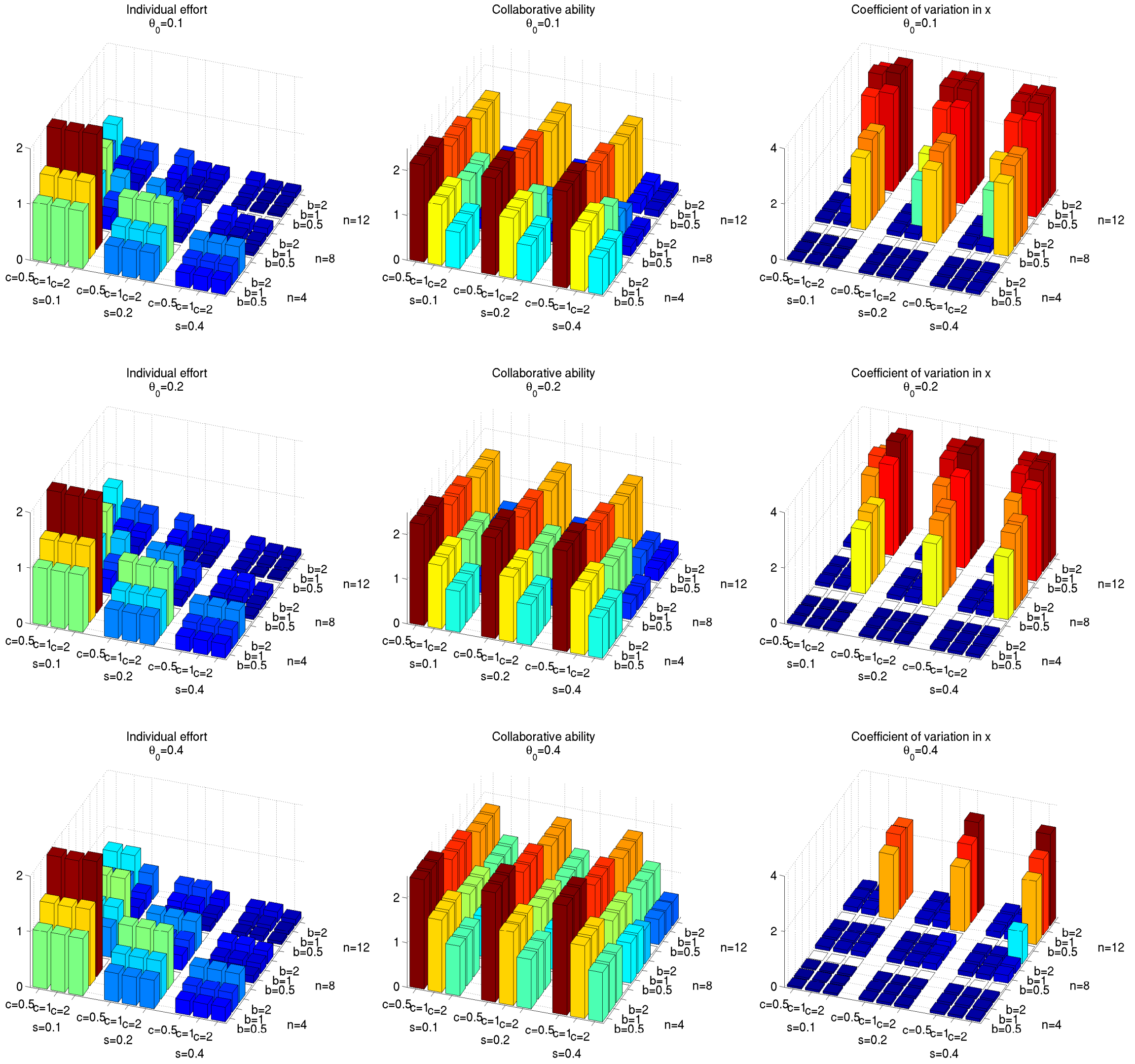

Figure 5: Collective action in “us vs. them” games.
